# A Comparison Study of Portable Foot-to-Foot Bioelectrical Impedance Scale to Measure Body Fat Percentage in Asian Adults and Children

**DOI:** 10.1155/2014/475659

**Published:** 2014-08-28

**Authors:** Pei Ying Sim, Tin Tin Su, Hazreen Abd Majid, Azmi Mohamed Nahar, Muhammad Yazid Jalaludin

**Affiliations:** ^1^Centre for Population Health (CePH) and Department of Social and Preventive Medicine, Faculty of Medicine, University of Malaya, 50603 Kuala Lumpur, Malaysia; ^2^Department of Sports Medicine, Faculty of Medicine, University of Malaya, 50603 Kuala Lumpur, Malaysia; ^3^Department of Paediatrics, Faculty of Medicine, University of Malaya, 50603 Kuala Lumpur, Malaysia

## Abstract

*Objective. *To compare the measurements of body fat percentage (BF%) using the foot-to-foot bioelectrical impedance analysis (FTF-BIA) with the direct segmental multifrequency BIA (DSM-BIA).* Methods. *There were 36 men and 52 women (37.1 ± 14.3 years) with 57% Malays, 30% Chinese, and 13% Indian. For children, there were 45 boys and 26 girls (11.5 ± 2.5 years) with 52% Malay, 15% Chinese, and 33% Indian.* Results. *Mean height for men was 168.4 cm, 11 cm taller than women. Men were 10 kg heavier than women at 70 kg. BF% in women was 32% and 33% whereas BF% in men was 23% and 25% when measured using FTF-BIA and DSM-BIA, respectively. In children, BF% measured with FTF-BIA and DSM-BIA was 49% and 46%, respectively. The correlations were significant for men (*r* = 0.92, SEE = 2.80), women (*r* = 0.91, SEE = 3.31), boys (*r* = 0.95, SEE = 5.44), and girls (*r* = 0.96, SEE = 5.27). The BF% in underweight/normal (*r* = 0.92, SEE = 2.47) and that in overweight/obese adults (*r* = 0.89, SEE = 3.61) were strongly correlated. The correlations were significant in normal/underweight (*r* = 0.94, SEE = 3.78) and obese/overweight children (*r* = 0.83, SEE = 6.49). All ethnic groups showed significant correlation with BF%. Malay adults (*r* = 0.92, SEE = 3.27) and children (*r* = 0.94, SEE = 0.88) showed significant mean differences in BF%.* Conclusion. *The FTF-BIA showed higher accuracy for all normal/underweight and Chinese group with acceptable overestimation in children and underestimation in adults. Caution should be taken when interpreting BF% depending on gender, BMI, and ethnicity.

## 1. Introduction

The bioelectrical impedance analysis (BIA) method is frequently used in research and clinical settings to measure body fat percentage (BF%) which is one of the important cardiovascular and metabolic risk factors in both children and adults [1–9]. There are two types of BIA methods: the foot-to-foot and the direct segmental multifrequency method. In the conventional foot-to-foot BIA method, there are four electrodes situated at each foot plate while the direct segmental multifrequency BIA (DSM-BIA) method has eight electrodes on each foot plate and hand handle. The foot-to-foot BIA method is more convenient in terms of portability and simplicity, and measurements are also reproducible [1]. The foot-to-foot BIA method can produce acceptable quantification of BF% with no significant differences in adults [2] and children [3, 4].

The DSM-BIA method measures impedance at five segments of the body (whole body, both feet and hands) by allowing the current and voltage to flow between hand and feet to quantify the BF%. The DSM-BIA has been compared as an acceptable tool for the quantification of BF% in comparison with the whole body dual-energy X-ray absorptiometry (DXA) scan in adults [5–7] and children [8]. The BF% values obtained from the other multiple-frequency BIA methods have also been cross-validated with the DXA scan in adults [4, 10–13] and children [11, 14, 15]. A recent study showed that foot-to-foot BIA overestimated BF% compared to DXA by 1.8% in athletic girls with mean age of 14 years [16]. Although the DXA is the gold standard method for measuring BF%, its use is limited to the clinical settings as the machine is bulky, nonportable, and expensive and requires technical expertise.

Because of the increasing trend in obesity and noncommunicable disease burden, it is necessary to measure accurate BF% rather than conventional BMI as a proxy measure of body fatness [10, 17]. In the low and middle income countries, there is an increasing demand for DSM-BIA in clinical settings such as the paediatric or adult obesity clinics, dietetic and sports medicine clinics. At the same time, foot-to-foot BIA is being widely used in large scale population based studies because of its reasonable quality, simple technology, and affordable price [18, 19]. These two commonly used methods to assess BF% in low and middle income countries should be compatible and comparable.

Ethnicity plays an important role and is highly associated with the BF% measured using the BIA method due to variations in the pattern of fat distribution [8, 20, 21]. The different body build among ethnic groups showed biasness in predicting body impedance which affected the validity of BIA [21]. The differences in BF% have been observed when comparing among white, black, Hispanic and Asian children [22]. Similarly, differences in BF% were also presented among Malay, Chinese and Indians [21]. A study conducted across South Africa and New Zealand comprising five ethnicities showed a direct association between body fat percentage and ethnicity [23]. The differences in body fat distribution across ethnic groups imply that ethnicity should be taken into account for the validity of body fat measurements.

To date, no studies have shown the correlations and limit of agreements between the foot-to-foot BIA and DSM-BIA methods in the Asian population. Majority of the BF% measurements using BIA are carried out in the Western population. Therefore, the aim of this study was to (1) compare the measurements of BF% using the foot-to-foot BIA with the DSM-BIA as the reference method in adults and children and (2) determine the effects of gender, BMI, and ethnicity on BF% measured using the foot-to-foot BIA in comparison with DSM-BIA in an Asian population.

## 2. Material and Methods

### 2.1. Subjects

Participants were recruited randomly in the morning from 9 am to 11 am from the paediatric and sports medicine clinics of University Malaya Medical Centre, Malaysia. Participants of the study were voluntary and no clinic staffs were involved in the recruitment process. The data collection was carried out from October to November 2013. All potential participants were wearing light clothing and no participants indicated they participated in strenuous activity or/and had diet treatments. Participants who did strenuous exercise or had diet treatments were excluded from the study. Before taking the measurements, participants were asked to remove all accessories and socks, empty their pockets, and also empty their bladder which could affect the electrical signals [24]. In total, 88 adults and 71 children met the inclusion criteria and were recruited for the study, whereby, there are 36 men, 52 women, 45 boys, and 26 girls.

Gender and age were recorded prior to the assessment of body composition. Participants were classified into either normal/underweight or obese/overweight groups and BMI was determined by weight (kg) divided by height (m) square. The BMI was obtained from the InBody370, Biospace, California, whole body bioelectrical impedance analyser which was used as the reference method in this study. The BMI cut-points for normal adults and children were 18.5 and 14.4 kgm^−2^, respectively, based on the International Obesity Task Force (IOTF) cut-offs [25]. Sociodemographic data such as age and gender were recorded. Of the 159 participants, there are 87 Malays, 37 Chinese, and 35 Indians. All participants consented and the study was approved by the Ethics Committee of the University Malaya Medical Centre.

### 2.2. Anthropometry

Height was measured using a wall-mounted stadiometer (SECA 780, Denver). Participants were advised to stand upright on each machine, with bare feet on the electrodes on the platform. Mean height of men and women was 168.4 (165.6, 171.1) cm and 157 (155, 158.8) cm, respectively, whereas for boys and girls the mean height was 11.5 (10.7, 12.2) cm and 11.2 (10.3, 12.2) cm, respectively. Data on weight and BF% were measured using 2 types of bioelectrical impedance analyser (BIA) machines: firstly the SC-240 Tanita, Tokyo, body composition analyser followed by the InBody370 whole body BIA. Height, gender, and age were entered into both the BIA systems prior to assessment. The measurement of SC-240 Tanita is based on the pressure contact on the footpad electrodes with a single frequency of 50–60 kHz. The BF% was determined based on the body impedance when a subthreshold electrical current passes through the body from leg to leg. These values are then compared to the reference values obtained from the InBody370 BIA which uses direct segmental multifrequency bioelectrical impedance analysis (DSM-BIA). The DSM-BIA method divides the human body into 5 sections by measuring impedance at the right arm, left arm, trunk, right leg, and left leg. Each section consists of tetrapolar 8-point tactile electrode measuring impedance with 3 different frequencies (5, 20, and 250 kHz) to estimate the intracellular and extracellular sections of the total body water. Aside from standing upright on the platform, participants were asked to grip the electrodes on the handles. Mean weight of men and women using the foot-to-foot BIA was 69.8 (64.5, 75.1) kg and 59.7 (55.8, 63.7) kg, respectively, whereas for boys and girls mean weight was 55.1 (48.6, 61.7) kg and 47.7 (38.0, 57.4) kg, respectively. When weight was measured using the DSM-BIA, the readings were as follows: men 70.2 (64.9, 75.4) kg, women 59.9 (55.9, 63.9) kg, boys 55.7 (49.0, 62.4) kg, and girls 48.0 (38.3, 57.7) kg.

### 2.3. Data Analyses

Analysis was carried out by age stratification—adults (mean age 37.1 ± 14.3 years) and children (mean age 11.5 ± 2.5 years) groups using IBM SPSS Statistical Package 21, Armonk, NY. Data were further divided into gender, BMI, and ethnicity. Means and 95% confidence intervals were calculated for all variables. The BF% measured by the DSM-BIA was used as the reference values. One-way analysis of variance was used to compare anthropometric data between gender, BMI groups, and ethnicity for adults and children.* t*-tests were used to compare the weight and BF% measured using the foot-to-foot BIA to the DSM-BIA. Simple linear regression analyses were carried out to investigate correlation between the reference DSM-BIA and foot-to-foot BIA. Furthermore, the limits of agreement in measurement of BF% between the 2 methods were assessed using the Bland and Altman plot [26]. The difference in BF% determined by foot-to-foot BIA and DSM-BIA was plotted against the average BF% obtained from the two BIA methods, represented in the Bland-Altman distribution. The mean differences between the two methods and the mean differences ±2 standard deviations of the difference between the methods were applied.

## 3. Results

A total of 159 participants (88 adults and 71 children) were recruited and the age range for children (boys and girls) was from 7 to 18 years and for adults (men and women) from 19 to 71 years (Table 1). In adults, men showed significantly higher age, height, and weight and lower BF% compared to the women but there was no significant difference in their BMI. In contrast, when comparing underweight/normal group with overweight/obese group, the age, weight, BMI, and BF% were significantly higher in the overweight/obese adults. However, the height, weight, BMI, and BF% were significantly higher in the overweight/obese group (Table 1). The BF% and weights were significantly different when comparing gender and BMI groups, regardless of the methods used.

Overall BF% in adults was underestimated by 7.2% for males and 3.3% for females when using the foot-to-foot BIA compared to the DSM-BIA. In children, the BF% was overestimated by 9.4% and 2.9% in boys and girls, respectively, with the foot-to-foot BIA compared to the DSM-BIA. Weights were only marginally lower when measured with the foot-to-foot BIA compared to DSM-BIA in both adults and children by 0.3% and 1%, respectively. Furthermore, BF% was underestimated by 3.8% for underweight/normal adults and 5.2% for overweight/obese adults when using the foot-to-foot BIA compared to the DSM-BIA. The BF% was underestimated by 4.8% in underweight/normal children but overestimated by 12.6% in overweight/obese children, with the foot-to-foot BIA compared to the DSM-BIA. However, there were no significant differences in the BF% and weight measured using the foot-to-foot BIA and DSM-BIA in both adults and children when separated by gender and BMI.

In Malay and Indian adults, BF% was underestimated by 7.2% and 2.9%, respectively, but the BF% in Chinese adults only showed an underestimation of 0.7% (Table 2) in the BF% measured by foot-to-foot BIA compared to the DSM-BIA. In BF% of children, Malay and Indian groups showed overestimation by 7.4% and 8.2%, respectively, and 1.5% in Chinese children in foot-to-foot BIA compared to DSM-BIA. In adults and children, BF% of Indian was the highest followed by Malay and Chinese ethnic groups, regardless of the methods used to measure BF%. The BF% of Indian children measured with foot-to-foot-BIA and DSM-BIA was significantly higher by 49% and 46%, respectively, compared to the Chinese children. Malay children showed a significantly higher BF% measured with DSM-BIA compared to the Chinese but no significant differences were observed in the adults (Table 2).

The correlation between BF% measured with the foot-to-foot BIA and DSM-BIA was significant (*P* < 0.001) for the adult population (*r* = 0.93, SEE = 3.66) and children population (*r* = 0.95, SEE = 4.18) (Table 3). When the population was separated by gender, the statistical significant correlation remained in both men (*r* = 0.92, SEE = 2.80) and women (*r* = 0.91, SEE = 3.31). In children, the correlation between the 2 measurements also showed statistical significance (*P* < 0.001) for boys (*r* = 0.95, SEE = 5.44) and girls (*r* = 0.96, SEE = 5.27). Furthermore, the foot-to-foot BIA formed significant correlation coefficients (*P* < 0.001) when the distribution was divided by BMI groups in both underweight/normal (*r* = 0.92, SEE = 2.47) and overweight/obese adults (*r* = 0.89, SEE = 3.61). In children, the significant correlations (*P* < 0.001) were presented but with a larger standard estimated error (SEE) in normal/underweight (*r* = 0.94, SEE = 3.78) and obese/overweight groups (*r* = 0.83, SEE = 6.49). As the population was separated by ethnicity, the statistical correlation was significant for adults in the Malay (*r* = 0.92, SEE = 0.85), Chinese (*r* = 0.93, SEE = 0.87), and Indian (*r* = 0.91, SEE = 0.83) group. Similarly in children, the Malay (*r* = 0.94, SEE = 0.88), Chinese (*r* = 0.98, SEE = 0.96), and Indian (*r* = 0.95, SEE = 0.89) group showed significant correlation between the two measurements.

**Figure 1:**
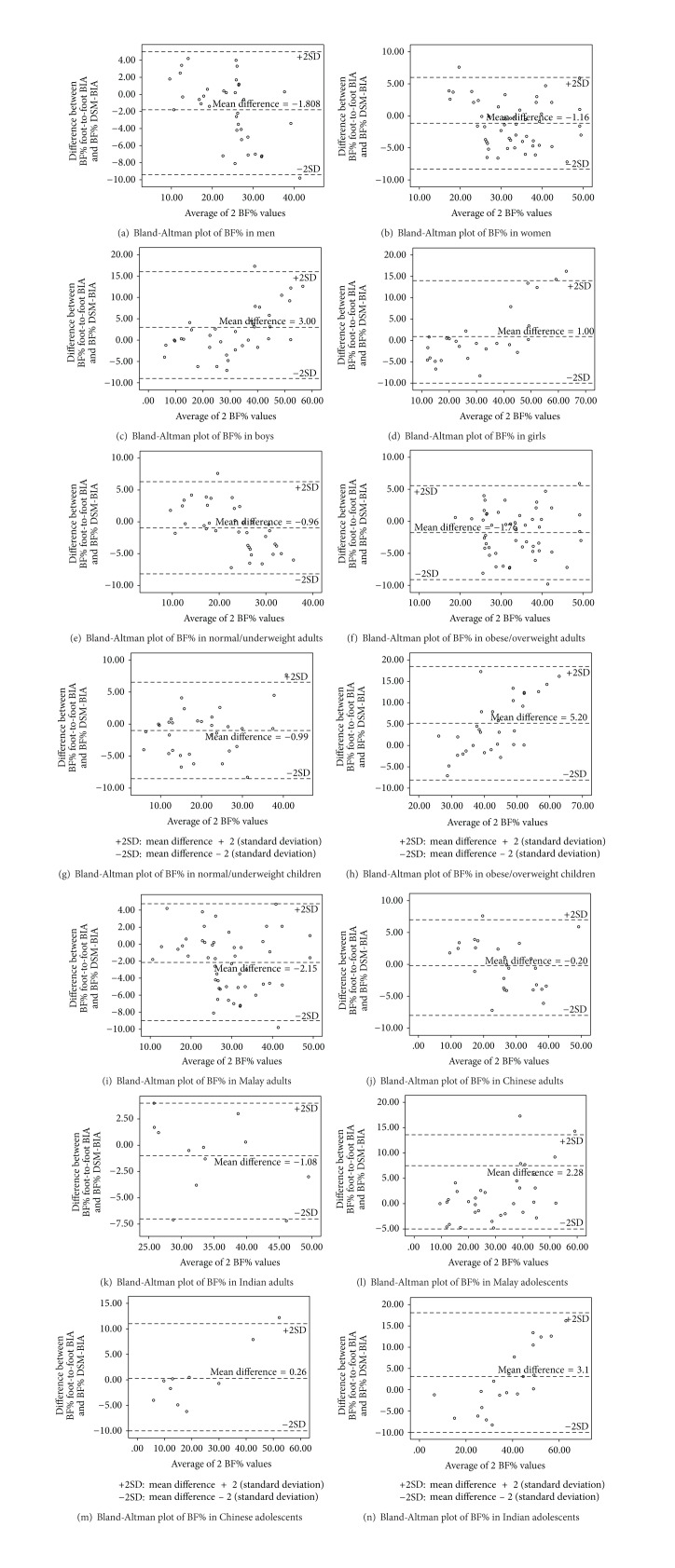
Bland-Altman plots of body fat percentage (BF%) in foot-to-foot BIA and direct segmental multifrequency BIA in adults ((a) men; (b) women) and children ((c) boys; (d) girls) by gender and in BMI groups adults ((e) normal/underweight; (f) obese/overweight) and children ((g) normal/underweight; (h) obese/overweight) and by ethnicity in adults ((i) Malay; (j) Chinese; (k) Indian) and children ((l) Malay; (m) Chinese; (n) Indian).

The Bland-Altman plots indicated no extreme over- or underestimation in the distribution of BF% using the foot-to-foot BIA for both adults and children by gender, BMI, and ethnicity groups (Figure 1). Mean differences for the foot-to-foot BIA when comparing with the DSM-BIA by gender were as follows: men −1.81 (−3.09, −0.53), women −1.16 (−2.15, −0.17), boys 2.99 (1.20, 4.80), and girls 0.91 (−1.76, 3.58). The foot-to-foot BIA significantly overestimated the BF% for 57% men (*P* < 0.05, Figure 1(a)) and underestimated 51% women (*P* < 0.05, Figure 1(b)) whereas, in children, 51% boys were significantly underestimated (*P* < 0.05) but the 69% girls with underestimated BF% did not vary significantly from the reference values (Figures 1(c) and 1(d)).

In addition, underestimation of BF% in 51% normal/underweight adults and overestimation of 54% in BF% of children did not significantly vary from the reference values. In normal/underweight and obese/overweight adults the mean differences were −0.96 (−2.17, 0.24) and −1.76 (−2.80, −0.73), respectively (Figures 1(e) and 1(f)). For normal/underweight and obese/overweight children, the mean differences were −0.99 (−2.30, 0.33) and 5.19 (2.97, 7.41), respectively (Figures 1(g) and 1(h)). However, the mean differences differed significantly from the reference values for the obese/overweight adults and children (*P* < 0.001) by an overestimation of BF% in 47% of the adults and underestimation of BF% in 51% of the children.

In adults, the mean differences were highest for Malay, −2.15 (−3.13, −1.17) (Figure 1(i)), followed by Indian, −1.08 (−3.37, 1.22) (Figure 1(k)), and Chinese, −0.20 (−1.73, 1.34) (Figure 1(j)), whereas in children the mean differences were highest for the Indian children, 3.10 (−0.15, 6.34) (Figure 1(n)), followed by the Malay, 2.28 (0.40, 4.17) (Figure 1(l)), and the Chinese, 0.26 (−3.37, 3.90) (Figure 1(m)). Of the three ethnic groups, only the Malay adults and children presented significant mean differences between the BF% measured with foot-to-foot BIA and DSM-BIA with an overestimation of BF% in 54% of the adults and underestimation of BF% in 86% of the children.

## 4. Discussion

This study showed that the BF% measured with foot-to-foot bioelectrical impedance analysis (BIA) was significantly and strongly correlated with measurements from the direct segmental multifrequency- (DSM-) BIA, using InBody370. In normal/underweight adults and children and in the girls, there were no significant differences in the measurement of BF% when compared to the reference method, DSM-BIA. This was in line with studies showing data on BF% in healthy/normal children when comparing with children who were HIV infected [14] and obese children with Down syndrome [15]. Additionally, a large study population of 591 healthy adults demonstrated that multifrequency BIA is a good alternative for quantifying BF% in healthy adults but leads to an underestimation in obese adults and overestimation in lean adults [4]. The BF% measured using the multifrequency BIA method showed that healthy adults with total BF% of >25% resulted in a significant but small underestimation of BF% by 4% when compared to the DXA scan [27].

Consistently, the BF% in adults was underestimated by 3.3% to 7.2% and BF% in children was overestimated by 2.9% to 9.4%. The underestimation of BF% in adults is common, as previous studies also showed underestimation in the adult population [4, 10, 28]. An overestimation of BF% by 3.0% was observed in boys and this was in parallel with studies showing overestimation of BF% in blacks [29], pubertal rural Chinese boys [30], and a large sample of 411 children aged 6 to 18 years [31].

Ethnicity is highly correlated with the BF% in both adults and children but more distinct mean differences among the ethnic groups were found in children. Indian Asians have been shown to develop higher BF% particularly abdominal fat compared to European and Pacific Island counterparts [32]. Similarly, in this study, Indians presented the highest BF% compared to the Malays and Chinese with a significant difference in children but not in adults. The BF% in different ethnic groups measured using the foot-to-foot BIA showed a consistent pattern of underestimation in adults and overestimation in children when compared to BF% measured using DSM-BIA with a negligible mean difference for the Chinese adults and children. Although the present study examined the BF% in a sample of multiethnic population of adults and children with significant mean differences in the Malay adults and children when using the two different methods, a larger sample is necessary to test the effects of ethnicity on the subjects.

It has been suggested that BIA is negatively affected by obesity [3, 27], particularly in women, thus underestimating the BF% [27, 33]. This was also observed in obese/overweight adults in this study whereby the underestimation of BF% in obese/overweight adults was as high as 5.2%. In contrast, a study by Shafer et al. [12] determined that the multiple-frequency BIA was a valid method for BF% with an underestimation of 1.6% in normal adults and an overestimation of 3.4% BF in obese adults in comparison with the DXA. In obese/overweight children, a significant overestimation of 12.6% was observed in this present study and this may be contributed by the overestimation of BF% from the reference method, as DSM-BIA has been previously shown to overestimate BF% by 0.3%–2.3% in children aged 10–17 years [11]. However, further investigation is required to support this finding.

The DSM-BIA method has been proven to have high reliability and accuracy [13] in healthy adults but less in women [5]. This did not tally with our study whereby a higher mean difference in BF% was measured in males compared to females between the foot-to-foot BIA and DSM-BIA. There were also no significant mean differences in girls and the explanation may be due to the higher BF% in boys causing a wider discrepancy between mean differences in BF%.

Overall, the Bland-Altman plots showed better agreements between the foot-to-foot BIA and reference DSM-BIA in the adults compared to the children. The scatter plots in the children, for all genders, obese/overweight, and all ethnic groups in children presented a linear relationship which showed the differences increased as the BF% increased. This indicates that the foot-to-foot BIA measurement for BF% in children needs to be interpreted with caution.

## 5. Conclusion

In conclusion, the wide limits of agreement in the measurement of BF% for the following groups, (i) men and boys, (ii) women, (iii) obese/overweight adults and children (both genders), and (iv) Malay adults and children, suggest that BIA is more appropriate in epidemiology studies rather than measures for self-assessment. Generally, there is a consistent pattern whereby the foot-to-foot BIA underestimates BF% in adults and overestimates BF% in children when compared to the DSM-BIA but with no significant differences. However, for group estimation, there is no extreme over- or underestimation in the distribution of BF%. Aside from the greater advantage of foot-to-foot BIA in terms of convenience, ease of measurement, and portability, BF% measured by foot-to-foot BIA is also strongly correlated with DSM-BIA. Therefore the foot-to-foot BIA is a suitable tool for community screening but caution should be taken when interpreting the BF% because it may be dependent on gender, BMI, and ethnic groups.

## Figures and Tables

**Table 1:** Descriptive characteristics of adults (*n* = 88) and children (*n* = 71) by gender and BMI groups.

Adults	Men (*n* = 36)	Women (*n* = 52)	Underweight or normal (*n* = 37)	Overweight or obese (*n* = 51)	Total (*n* = 88)
Age (years)	41.7 (36.7, 46.7)	33.5 (29.8, 37.2)∗	30.0 (25.9, 34.0)	41.8 (37.9, 45.7)∗∗	36.8 (33.8, 39.9)
Height (cm)	168.4 (165.6, 171.1)	157.1 (155.3, 158.8)∗∗	161.7 (159.0, 164.3)	161.7 (159.0, 164.5)	161.7 (159.8, 163.6)
Weight by foot BIA (kg)	69.8 (64.5, 75.1)	59.7 (55.8, 63.7)∗	52.7 (49.2, 56.1)	72.0 (68.1, 75.8)∗∗	63.9 (60.6, 67.2)
Weight by DSM-BIA (kg)	70.2 (64.9, 75.4)	59.9 (55.9, 63.9)∗	53.0 (49.6, 56.3)	72.2 (68.3, 76.0)∗∗	64.1 (60.8, 67.4)
Body mass index (kg/m^2^)	25.1 (23.9, 26.3)	24.3 (22.8, 25.7)	20.7 (20.1, 21.2)	27.5 (26.4, 28.6)∗∗	24.6 (23.6, 25.6)
Body fat foot BIA (%)	23.2 (20.9, 25.6)	32.3 (30.0, 34.5)∗∗	22.6 (20.5, 24.6)	32.9 (30.7, 35.1)∗∗	28.6 (26.7, 30.4)
Body fat by DSM-BIA (%)	25.0 (22.0, 28.1)	33.4 (31.0, 35.9)∗∗	23.5 (20.8, 26.3)	34.7 (32.5, 36.9)∗∗	30.0 (28.0, 32.1)

Children	Boys (*n* = 45)	Girls (*n* = 26)	Underweight or normal (*n* = 34)	Overweight or obese (*n* = 37)	Total (*n* = 71)

Age (Years)	11.5 (10.7, 12.2)	11.2 (10.3, 12.2)	11.4 (10.5, 12.2)	11.4 (10.5, 12.3)	11.4 (10.8, 12.0)
Height (cm)	150.0 (145.3, 154.8)	145.1 (140.2, 149.9)	144.0 (139.4, 148.6)	152.1 (147.1, 157.1)∗	148.2 (144.7, 151.7)
Weight by foot BIA (kg)	55.1 (48.6, 61.7)	47.7 (38.0, 57.4)	36.0 (32.5, 39.5)	67.5 (60.6, 74.3)∗∗	52.4 (47.0, 57.8)
Weight by DSM-BIA (kg)	55.7 (49.0, 62.4)	48.0 (38.3, 57.7)	36.3 (32.8, 39.8)	68.1 (61.1, 75.1)∗∗	52.9 (47.4, 58.3)
Body mass index (kg/m^2^)	24.5 (22.5, 26.5)	21.9 (18.5, 25.3)	17.2 (16.2, 18.3)	29.3 (27.5, 31.1)∗∗	23.5 (21.8, 25.3)
Body fat foot BIA (%)	34.8 (29.8, 39.8)	31.9 (24.5, 39.3)	19.8 (16.2, 23.5)	46.5 (42.7, 50.3)∗∗	33.7 (29.6, 37.8)
Body fat by DSM-BIA (%)	31.8 (27.8, 35.7)	31.0 (25.5, 36.5)	20.8 (17.6, 24.1)	41.3 (38.8, 43.8)∗∗	31.5 (28.4, 34.6)

Means (95% confidence intervals) were presented. *P < 0.05; **P < 0.001 tested with analysis of variance within groups.

**Table 2:** Descriptive characteristics of adults (*n* = 88) and children (*n* = 71) by ethnicity.

Adults	Malay (*n* = 50)	Chinese (*n* = 26)	Indian (*n* = 12)	*P* value
Age (years)	34.9 (31.5, 38.2)	39.7 (33.0, 46.4)	38.8 (26.9, 50.6)	0.34
Height (cm)	160.8 (158.4, 163.3)	163.8 (160.2, 167.4)	160.6 (153.9, 167.3)	0.36
Weight by foot BIA (kg)	62.6 (58.1, 67.2)	63.7 (57.2, 70.3)	69.3 (61.1, 77.4)	0.42
Weight by DSM-BIA (kg)	62.9 (58.4, 67.3)	63.9 (57.4, 70.4)	69.7 (61.7, 77.7)	0.39
Body mass index (kg/m^2^)	24.6 (23.2, 25.9)	23.6 (21.8, 25.5)	26.9 (24.7, 29.1)	0.12
Body fat foot BIA (%)	28.3 (25.9, 30.7)	26.7 (22.9, 30.4)	33.8 (29.1, 38.3)	0.06
Body fat by DSM-BIA (%)	30.5 (27.9, 33.1)	26.9 (22.6, 31.1)	34.8 (29.3, 40.4)	0.05

Children	Malay (*n* = 37)	Chinese (*n* = 11)	Indian (*n* = 23)	*P* value

Age (years)	10.8 (10.0, 11.6)	12.0 (10.3, 13.7)	12.0 (11.0, 13.0)	0.15
Height (cm)	146.7 (141.5, 152.0)	147.3 (137.3, 157.2)	151.0 (145.5, 156.5)	0.55
Weight by foot BIA (kg)	52.0 (44.0, 60.0)	39.9 (28.0, 51.9)	59.0 (50.0, 68.0)	0.07
Weight by DSM-BIA (kg)	52.3 (44.3, 60.4)	40.1 (28.2, 52.1)	59.9 (50.6, 69.1)	0.06
Body mass index (kg/m^2^)	23.7 (21.2, 26.2)^a^	17.8 (14.4, 21.2)^b^	26.0 (22.9, 29.1)^a^	<0.05
Body fat foot BIA (%)	33.2 (28.1, 38.3)^a,b^	20.8 (9.3, 32.3)^a^	40.8 (33.4, 48.2)^b^	<0.05
Body fat by DSM-BIA (%)	30.9 (26.7, 35.1)^a^	20.5 (12.0, 29.0)^b^	37.7 (32.9, 42.5)^a^	<0.05

Means (95% confidence intervals) were presented. *P* values were obtained from ANOVA with Tukey's multiple post hoc testing. Means with different subscripts indicate significant difference and vice versa. NS: nonsignificant.

**Table 3:** Methodological correlations of foot-to-foot BIA against direct segmental multifrequency BIA reference method for body fat percentage in adults (*n* = 88) and children (*n* = 71).

	Adults (*n* = 88)	*r*	*r* ^2^*l*^^	SEE	Total error, *E*	Mean differences	Slope	Intercept	Standardized coefficient
	All	0.926∗∗	0.858	3.662	3.112	0.58 (−1.89, 3.43)	0.77	1.02	0.926
Gender	Men	0.916∗∗	0.839	2.795	3.192	−1.81 (−3.09, −0.53)∗	1.19	−2.52	2.499
	Women	0.913∗∗	0.833	3.309	3.117	−1.16 (−2.15, −0.17)∗	0.99	1.46	2.492
BMI groups	Normal/underweight	0.916∗∗	0.840	2.465	3.030	−0.96 (−2.17, 0.24)	1.24	4.5	2.499
	Obese/overweight	0.889∗∗	0.790	3.610	3.233	−1.76 (−2.80, −0.73)	0.89	5.51	2.433
Ethnicity	Malay	0.924∗∗	0.854	3.265	3.208	−2.15 (−3.13, −1.17)∗∗	0.87	1.94	0.924
	Chinese	0.933∗∗	0.870	3.409	3.204	−0.20 (−1.73, 1.34)	0.83	4.48	0.933
	Indian	0.913∗∗	0.833	3.118	2.780	−1.08 (−3.37, 1.22)	0.76	7.12	0.913

	Children (*n* = 71)	*r*	*r* ^2^*l*^^	SEE	Total error, *E*	Mean differences	Slope	Intercept	Standardized coefficient

	All	0.949∗∗	0.902	4.183	4.735	6.31 (4.73, 9.11)∗∗	0.73	6.92	0.949
Gender	Boys	0.946∗∗	0.895	5.440	4.811	2.99 (1.20, 4.80)∗	0.75	5.71	2.575
	Girls	0.96∗∗	0.921	5.268	4.603	0.91 (−1.76, 3.58)	0.71	8.48	2.612
BMI groups	Normal/underweight	0.935∗∗	0.874	3.782	2.885	−0.99 (−2.30, 0.33)	0.82	4.51	2.547
	Obese/overweight	0.825∗∗	0.681	6.493	6.423	5.19 (2.97, 7.41)∗∗	0.55	15.83	2.282
Ethnicity	Malay	0.936∗∗	0.877	5.453	4.111	2.28 (0.40, 4.17)∗	1.14	2.01	0.936
	Chinese	0.979∗∗	0.959	3.653	3.518	0.26 (−3.37, 3.90)	1.33	6.48	0.979
	Indian	0.946∗∗	0.894	5.699	6.322	3.10 (−0.15, 6.34)	1.45	−13.88	0.946

Mean differences are presented in mean (95% confidence interval). *r*: correlation coefficient between BF% measured with foot-to-foot BIA and reference method; *r* ^2^ indicates how well the BF% measured with foot-to-foot BIA fits the goodness of fit line; SEE: standardized estimated errors; *E*: total error; *P < 0.05; **P < 0.001.
